# Mesencephalic origin of the inferior lobe in zebrafish

**DOI:** 10.1186/s12915-019-0631-y

**Published:** 2019-03-08

**Authors:** Solal Bloch, Manon Thomas, Ingrid Colin, Sonya Galant, Elodie Machado, Pierre Affaticati, Arnim Jenett, Kei Yamamoto

**Affiliations:** 1 Paris-Saclay Institute of Neuroscience (Neuro-PSI), CNRS UMR9197, Univ Paris Sud, Université Paris-Saclay, CNRS Bâtiment 5, Avenue de la Terrasse, 91190 Gif-sur-Yvette, France; 2 TEFOR Paris-Saclay, CNRS UMS2010, INRA UMS1451, Univ Paris Sud, Université Paris-Saclay, 91190 Gif-sur-Yvette, France; 3 Present address: Plateau de phénotypage TEFOR, LPGP-INRA UR1037, 35042 Rennes, France

**Keywords:** Teleost, Midbrain, Forebrain, Evolution, Homology, Vertebrate, Comparative neuroanatomy, Development, Cell lineage, Ventricle

## Abstract

**Background:**

Although the overall brain organization is shared in vertebrates, there are significant differences within subregions among different groups, notably between Sarcopterygii (lobe-finned fish) and Actinopterygii (ray-finned fish). Recent comparative studies focusing on the ventricular morphology have revealed a large diversity of the hypothalamus. Here, we study the development of the inferior lobe (IL), a prominent structure forming a bump on the ventral surface of the teleost brain. Based on its position, IL has been thought to be part of the hypothalamus (therefore forebrain).

**Results:**

Taking advantage of genetic lineage-tracing techniques in zebrafish, we reveal that cells originating from *her5*-expressing progenitors in the midbrain-hindbrain boundary (MHB) participate in the formation of a large part of the IL. 3D visualization demonstrated how IL develops in relation to the ventricular system. We found that IL is constituted by two developmental components: the periventricular zone of hypothalamic origin and the external zone of mesencephalic origin. The mesencephalic external zone grows progressively until adulthood by adding new cells throughout development.

**Conclusion:**

Our results disprove a homology between the IL and the mammalian lateral hypothalamus. We suggest that the IL is likely to be involved in multimodal sensory integration rather than feeding motivation. The teleost brain is not a simpler version of the mammalian brain, and our study highlights the evolutionary plasticity of the brain which gives rise to novel structures.

**Electronic supplementary material:**

The online version of this article (10.1186/s12915-019-0631-y) contains supplementary material, which is available to authorized users.

## Background

The vertebrate brain is considered to be divided into three main domains: the forebrain (prosencephalon), the midbrain (mesencephalon), and the hindbrain (rhombencephalon). Brain morphogenesis at early embryonic stages is controlled by local signaling centers called the “organizers.” Different organizers are set up successively over time during early development, with the primary organizer being fundamental for the primary neural induction. By interacting with transcription factors, the secondary organizers such as the anterior neural ridge (ANR) and the *zona limitans intrathalamica* (ZLI) control the morphogenesis of the forebrain, while the isthmic organizer (IsO) located at the midbrain-hindbrain boundary (MHB) controls the morphogenesis of the midbrain and the anterior hindbrain (reviewed in [1]).

Compared to the large morphological diversity in adulthood, embryonic brains appear relatively similar among different vertebrate groups. For this reason, anatomical comparison at this stage is helpful to understand the basic arrangement of the brain morphology. It is accepted that the brain regionalization depends on the establishment of subdivisions along the anterior-posterior (A-P) and dorsal-ventral (D-V) axes of the neural tube. Furthermore, the neuromeric model has refined this view by introducing the notion of segmentation unit along these axes [2, 3].

**Fig. 1:**
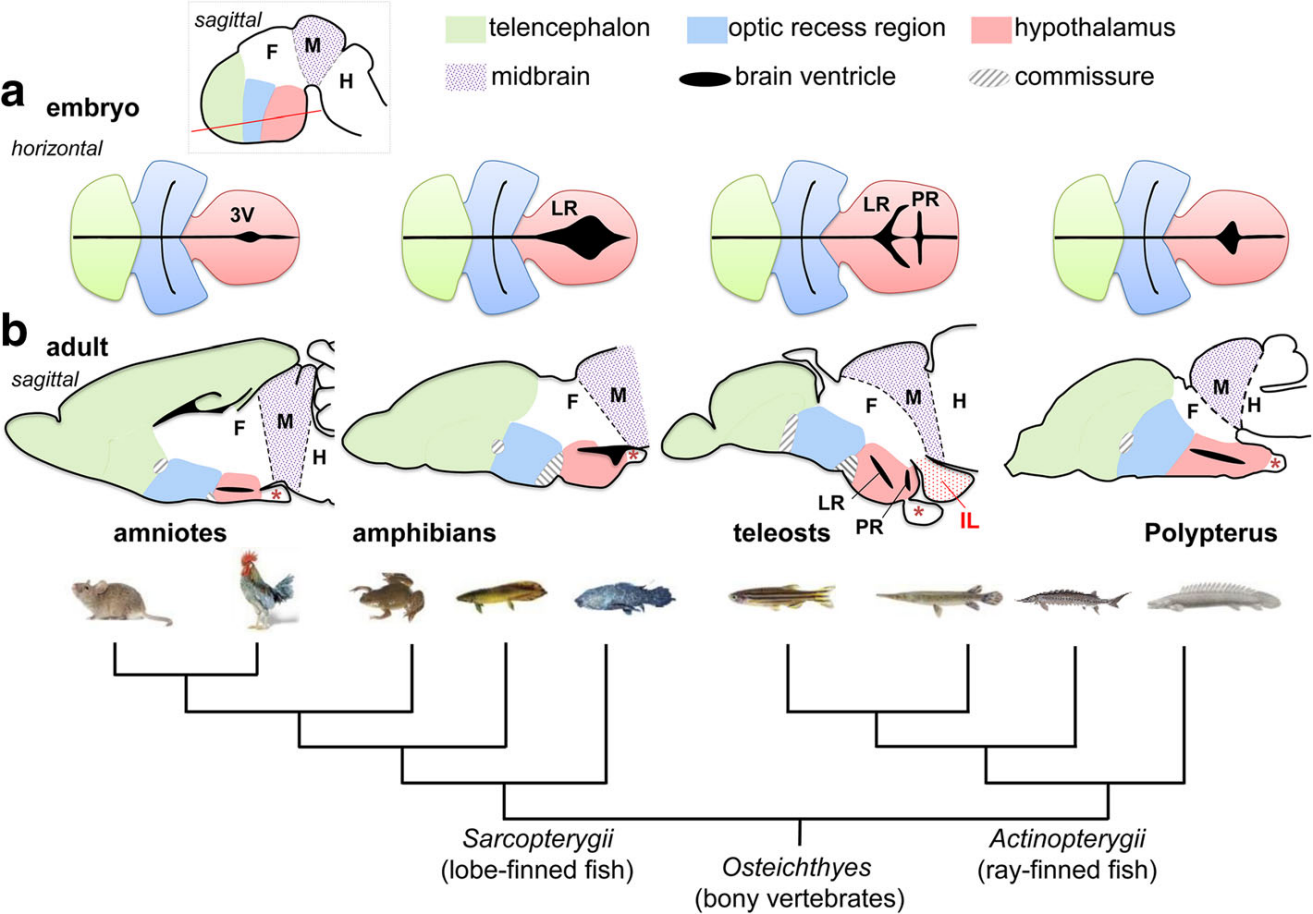
Evolution of the hypothalamic ventricles in bony vertebrates. Schematic drawing of embryonic (**a**) and adult (**b**) brains of rodent, frog, zebrafish, and *Polypterus* is shown above a phylogenetic tree of the *Osteichthyes* (bony vertebrates). **a** Horizontal view of embryonic brains through the anterior forebrain, highlighting the morphological diversity of the hypothalamic recesses (ventricular zones are shown in black). The horizontal section level (red line) is displayed in the top left panel (dotted square) with in a schematic embryonic brain. **b** Sagittal sections of adult brains. The inferior lobe (IL) is a brain structure present in teleosts but not in tetrapods or in *Polypterus*. The asterisks indicate hypophysis in each animal. Abbreviations: 3 V third ventricle, F forebrain, H hindbrain, IL inferior lobe, LR lateral recess, M midbrain, PR posterior recess

Regarding regional subdivisions of the most anterior part of the forebrain (secondary prosencephalon), modification of the current prosomeric model has been proposed [4–6]: based on the morphological analysis focusing on the ventricular organization, the secondary prosencephalon is divided into three subdivisions (Fig. 1), the telencephalon, the hypothalamus, and the optic recess region (ORR) that extends laterally to form the primordium of the retina [4, 5]. This new view redefines the boundary of the “hypothalamus.” For example, the “alar hypothalamus” of the prosomeric model in tetrapods containing neuroendocrine cells appears to be part of the ORR.

The analysis focusing on the ventricular organization also sheds light on the diversity of the hypothalamic morphology in *Osteichthyes* (bony vertebrates) [7]. In mammals and birds (amniotes), the hypothalamic ventricle is thin and indistinguishable from the diencephalic part of the third ventricle (3V, Fig. 1a, amniotes). In amphibians, the 3V is extended laterally and thus it is named “the lateral recess (LR) of the infundibulum” [8] (LR; Fig. 1a, amphibians). In teleosts, the hypothalamic ventricle is larger and more complex. In addition to the lateral recess (LR), which corresponds to the tetrapod 3V, teleosts have another recess named the posterior recess (PR; Fig. 1a, b, teleosts). Furthermore, the teleost LR is extremely elongated in the mature brain surrounding the PR, and it forms an additional structure named the “inferior lobe of the hypothalamus” (IL; Fig. 1b, teleosts, red dotted area) [9, 10]. The IL is a structure, which is not present in tetrapods, likely to have evolved specifically in a certain group of Actinopterygii that contains teleosts and holosteans (including gars; Lepisosteidae).

In this study, we examined the developmental origin of the IL in the zebrafish brain, by taking advantage of a cell lineage method based on tamoxifen inducible Cre-lox recombination. It revealed that the external zone of the IL is composed by the progeny of *her5*-expressing MHB cells, suggesting that a large part of the IL is actually of mesencephalic origin. Inductions at different time points during development provided further information on how this structure forms.

## Results

### Distribution of cells originating from the 24 hpf MHB in the adult brain

**Fig. 2:**
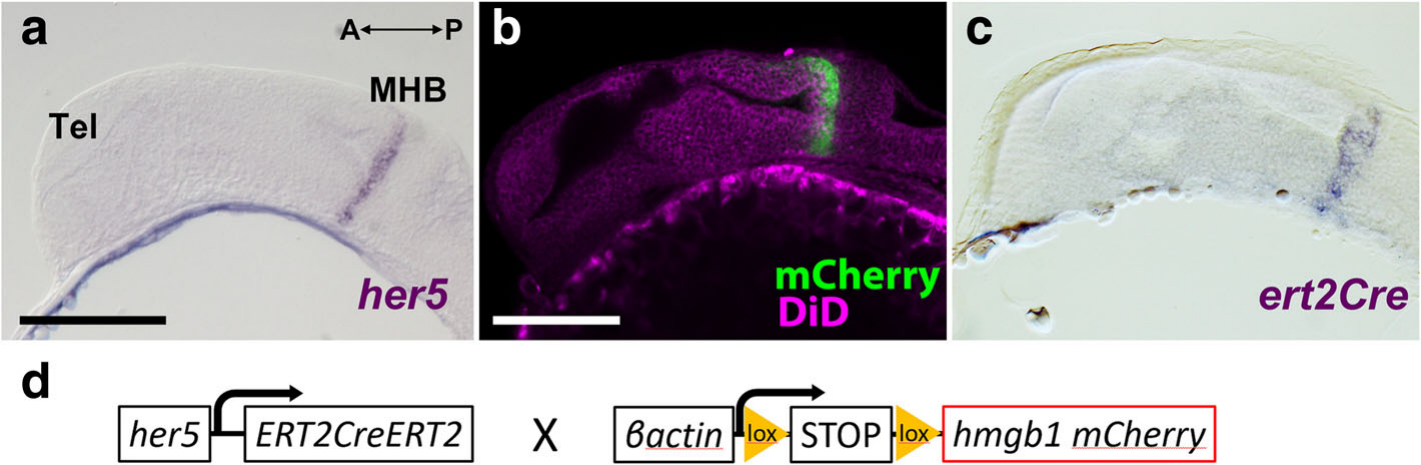
Verification of the expression profile of the zebrafish transgenic line used in this study. **a**–**c** Sagittal sections of 24 hpf embryos showing that expression of *her5* and *ert2Cre* is restricted to the MHB. The anterior part of the embryo is on the left. **a** In situ hybridization (ISH) of *her5* confirms its specific expression in the MHB (purple). **b** Expression of mCherry (green) in a transgenic line *Tg(her5:mCherry)*, which is identical to the *her5* ISH pattern. The morphology is shown with DiD fiber labeling (magenta). **c** The expression pattern of *ert2Cre* is also identical to the *her5* ISH (**a**) and mCherry in *Tg(her5:mCherry)* (**b**). **d** A simplified schema of the constructs of *Tg(her5:ERT2CreERT2)* and *Tg(βact:lox-stop-lox-hmgb1:mCherry)* which were used in this cell lineage study. Scale bars, 100 μm. Abbreviations: MHB midbrain-hindbrain boundary, Tel telencephalon


The *her5* transcription factor is known to be an early marker of the midbrain-hindbrain domain in zebrafish [11, 12]. Indeed, the zebrafish MHB is well established at 24 hours post-fertilization (hpf), and at this stage, *her5* expression is restricted to the MHB ([13]; see also Fig. 2a, b, Additional file 1: Movie S1.). In sagittal sections, the *her5* expression territory appears as a thin band, extending from dorsal to ventral. To follow the fate of these *her5*-expressing progenitors, we crossed two transgenic lines: *Tg(her5:ERT2CreERT2)* and *Tg(βact:lox-stop-lox-hmgb1:mCherry)* (Fig. 2d). We verified that the Cre expression is limited to the MHB at 24 hpf, recapitulating the *her5* expression (Fig. 2c). Thus, after tamoxifen treatment at 24 hpf, cells expressing *her5* and their progenies express mCherry, and we can interpret that all the mCherry-positive cells observed at later stages (after induction) are derived from the MHB.

Additional file 1:**Movie S1.** Expression of *her5* in the 24 hpf zebrafish embryo. Expression of mCherry in a transgenic line *Tg(her5:mCherry)* is shown in magenta, and DiD fiber labeling indicating the morphology of the embryo is shown in gray. (MP4 1999 kb)


**Fig. 3:**
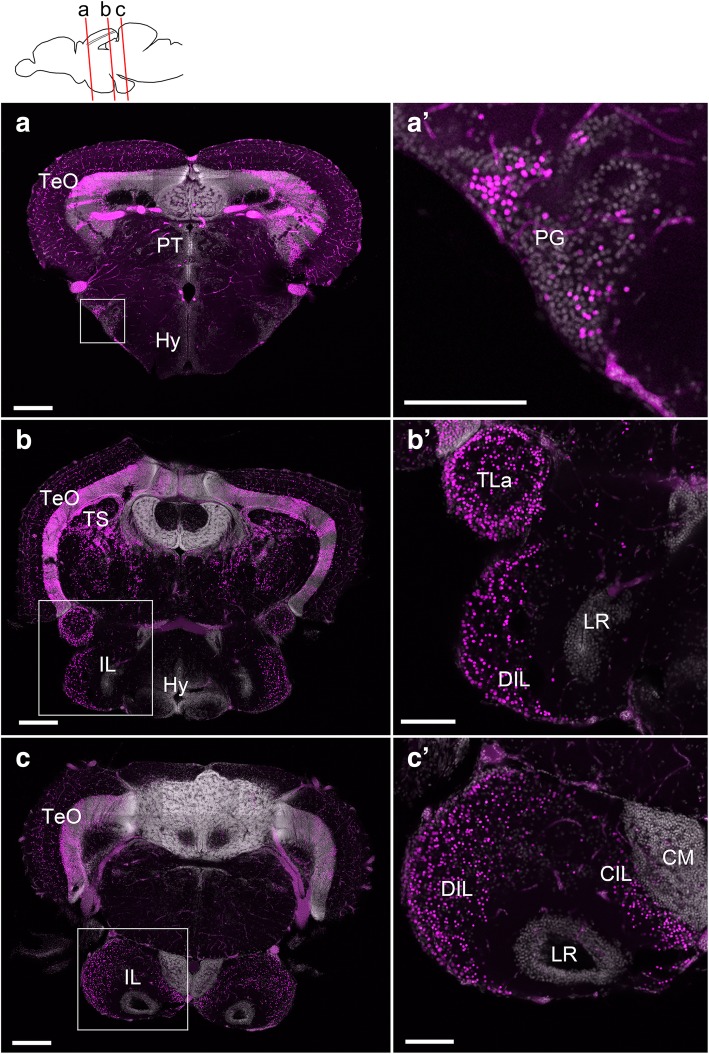
Localization of the mCherry-positive cells in the adult brain of *Tg(her5:ERT2CreERT2;βact:lox-stop-lox-hmgb1:mCherry)* zebrafish treated with tamoxifen at 24 hpf. **a**–**c** Confocal images of frontal sections showing global views of the mCherry distribution (*Z*-projection, 5 μm for **a** and 10 μm for **b** and **c**). The mCherry-positive cells are shown in magenta, and DAPI nuclear labeling is shown in gray. The plane of each section is indicated in the schematic drawing on the top. **a’**–**c’** Higher magnifications of the areas squared in **a**–**c**, showing the preglomecular nucleus (PG; **a’**) and the inferior lobe (IL; **b’**, **c’**). Abbreviations: CM corpus mamillare, CIL central nucleus of the inferior lobe, DIL diffuse nucleus of the inferior lobe, Hy hypothalamus, IL inferior lobe, LR lateral recess, PG preglomerular nucleus, PT pretectum, TeO optic tectum, TLa torus lateralis, TS torus semicircularis. Scale bars: **a**–**c**, 200 μm; **a’**–**c’**, 100 μm

As expected, in the brains of adult fish (3 months or older), mesencephalic structures such as the tectum (TeO), the torus semicircularis (TS), and the tegmental area were massively mCherry positive after induction with tamoxifen at 24 hpf (Fig. 3a–c). There was no labeling in forebrain structures such as the hypothalamus or the pretectum (Hy and PT respectively; Fig. 3a, b).

However, some unexpected structures exhibited mCherry-positive cells. The preglomerular nucleus (PG; Fig. 3a’) is a sensory relay nuclear complex that is considered to be part of the posterior tuberculum of the diencephalon. The PG is continuous with a nucleus named the torus lateralis (TLa; Fig. 3b). TLa is classified as part of the tegmentum (thus midbrain) by some authors [14, 15], while as a diencephalic (thus forebrain) by others [10, 16]. The cluster of mCherry-positive cells is continuous from PG to IL through TLa.

The IL is usually considered as part of the hypothalamus [14] because of its location posterior to the hypothalamus proper. Indeed, this structure develops as a lateral elongation of the teleost LR (which corresponds to the amniote 3V in the hypothalamus). However, our data showing a massive of mCherry-positive cells in IL (Fig. 3b, c, Additional file 2: Figure S1) suggest that a large part of this structure is formed by cells originating from the MHB. The diffuse nucleus of the inferior lobe [10] (DIL; Fig. 3b’, c’) is the most labeled area. Numerous mCherry-positive cells are also found in the area corresponding to the central nucleus of the inferior lobe (CIL; Fig. 3c’). Interestingly, most of the outer region of the IL is mCherry positive, but the inner part, close to the LR, is mCherry-negative (Fig. 3b’, c’). The dorso-medial structure called the corpus mamillare (CM) is also mCherry-negative (Fig. 3c’).

### Migration of the cells originating from the MHB during development

Our data on the adult brain show that IL has a mesencephalic component. In order to decipher the development and anatomy of this structure in further details, we followed the mCherry-positive cells in whole brains at different stages after induction at 24 hpf.

**Fig. 4:**
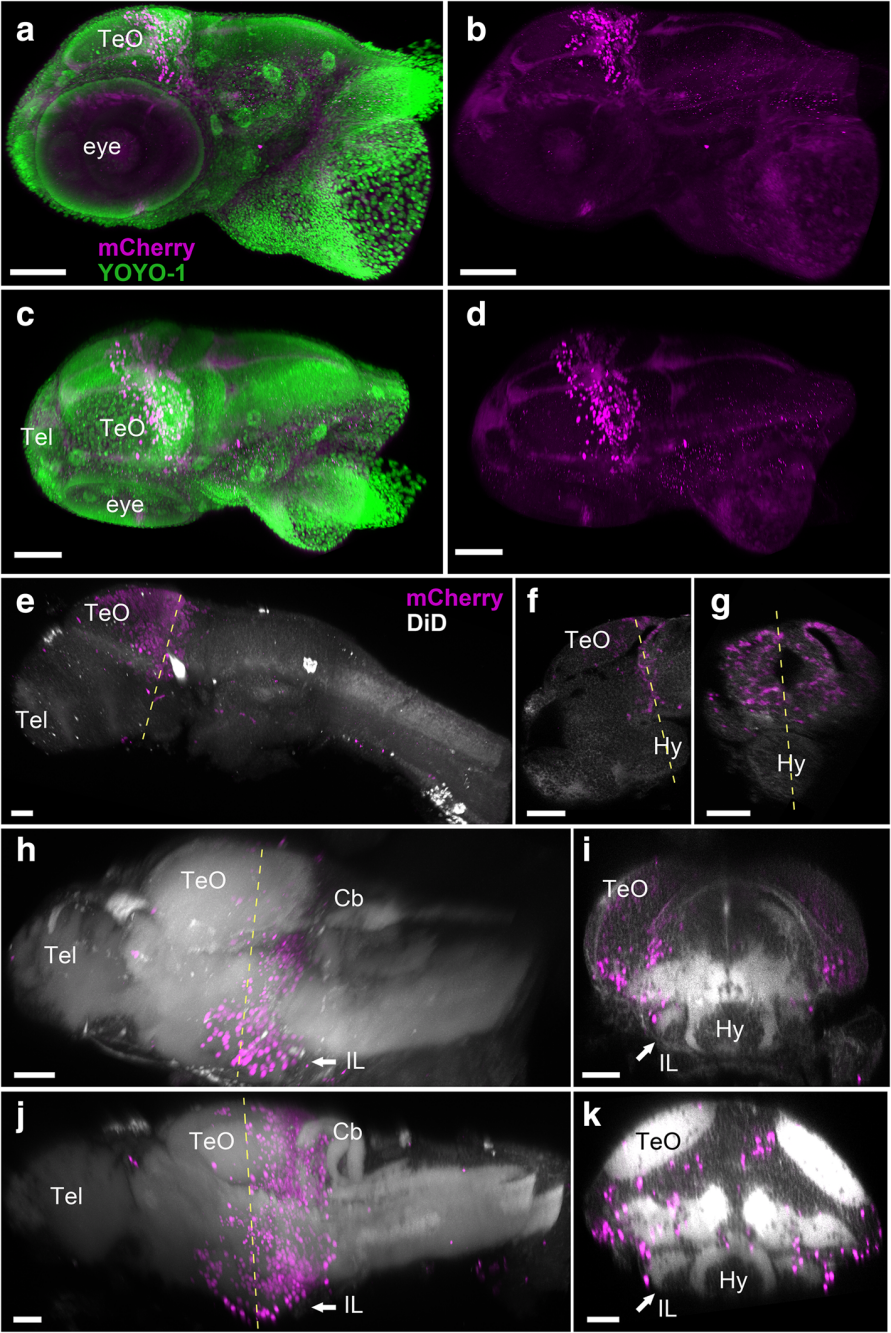
Localization of the mCherry-positive cells in young larval brains of *Tg(her5:ERT2CreERT2;βact:lox-stop-lox-hmgb1:mCherry)* zebrafish treated with tamoxifen at 24 hpf. Anterior to the left for **a**–**d**, **e**, **h**, and **j**. **a**–**d** 3D reconstruction from confocal images of a whole head of 3 dpf larva. mCherry-positive cells are shown in magenta, and YOYO-1, a nuclear marker, is shown in green. **a**, **b** Side view of the larval head with (**a**) and without (**b**) YOYO-1 labeling. **c**, **d** Top view of the larval head with (**c**) and without (**d**) YOYO-1 labeling. The mCherry-positive cells are still close to the MHB at this stage. Some cells are starting to migrate anteriorly, but there are no mCherry-positive cells in the forebrain or in other brain areas. **e**–**k** 3D reconstruction from confocal images of dissected brains of 3 dpf (**e**–**g**), 5 dpf (**h**, **i**), and 7 dpf (**j**, **k**) larvae. mCherry-positive cells are shown in magenta, and DiD fiber labeling is shown in gray. **e** A whole brain at 3 dpf is shown in lateral view. **f** A sagittal section through the same specimen. **g** A frontal section. The hypothalamus (Hy) is extending in ventral position below the midbrain and is devoid of mCherry-positive cells. **h** A whole brain at 5 dpf is shown in lateral view. **i** A frontal section from the same brain showing the first appearance of the inferior lobe (IL; arrow), with a few mCherry-positive cells at the periphery of the structure. **j** A whole brain at 7 dpf is shown in lateral view. **k** A frontal section from the same brain showing the growing IL (arrow), with more mCherry-positive cells added laterally. Abbreviations: Cb cerebellum, Hy hypothalamus, IL inferior lobe, TeO optic tectum, Tel telencephalon. Scale bars: **a**–**d**, 100 μm. **e**–**k**, 50 μm


At 3 days post-fertilization (dpf), all the mCherry-positive cells are still located around the MHB, and there is no labeling at the level of the forebrain (Fig. 4a–g, Additional file 3: Movie S2). This confirms the specificity of the molecular cell tracing method and the absence of leaky expression during the induction process. It further supports that the anterior structures, in which we observe mCherry expression at later stages, are composed of the progeny of cells originating from the MHB exclusively.

Additional file 3:**Movie S2.** Localization of the mCherry-positive cells in the 3 dpf larval head of *Tg(her5:ERT2CreERT2;βact:lox-stop-lox-hmgb1:mCherry)* zebrafish treated with tamoxifen at 24 hpf (3D visualization of Fig. 4a–d). mCherry-positive cells are shown in magenta, and YOYO-1, a nuclear marker, is shown in green. (MP4 5759 kb)


At 3 dpf, we could not identify the IL. In the lateral view (Fig. 4e), the MHB progenies (cells which were expressing *her5* at 24 hpf) extend in a triangular cluster, wider in the dorsal part. The IL was first observable at 5 dpf (Fig. 4h, i; arrows), being more remarkable at 7 dpf (Fig. 4j, k; arrows). MHB progenies extend ventrally in the outer surface of IL (Fig. 4i, k).

**Fig. 5:**
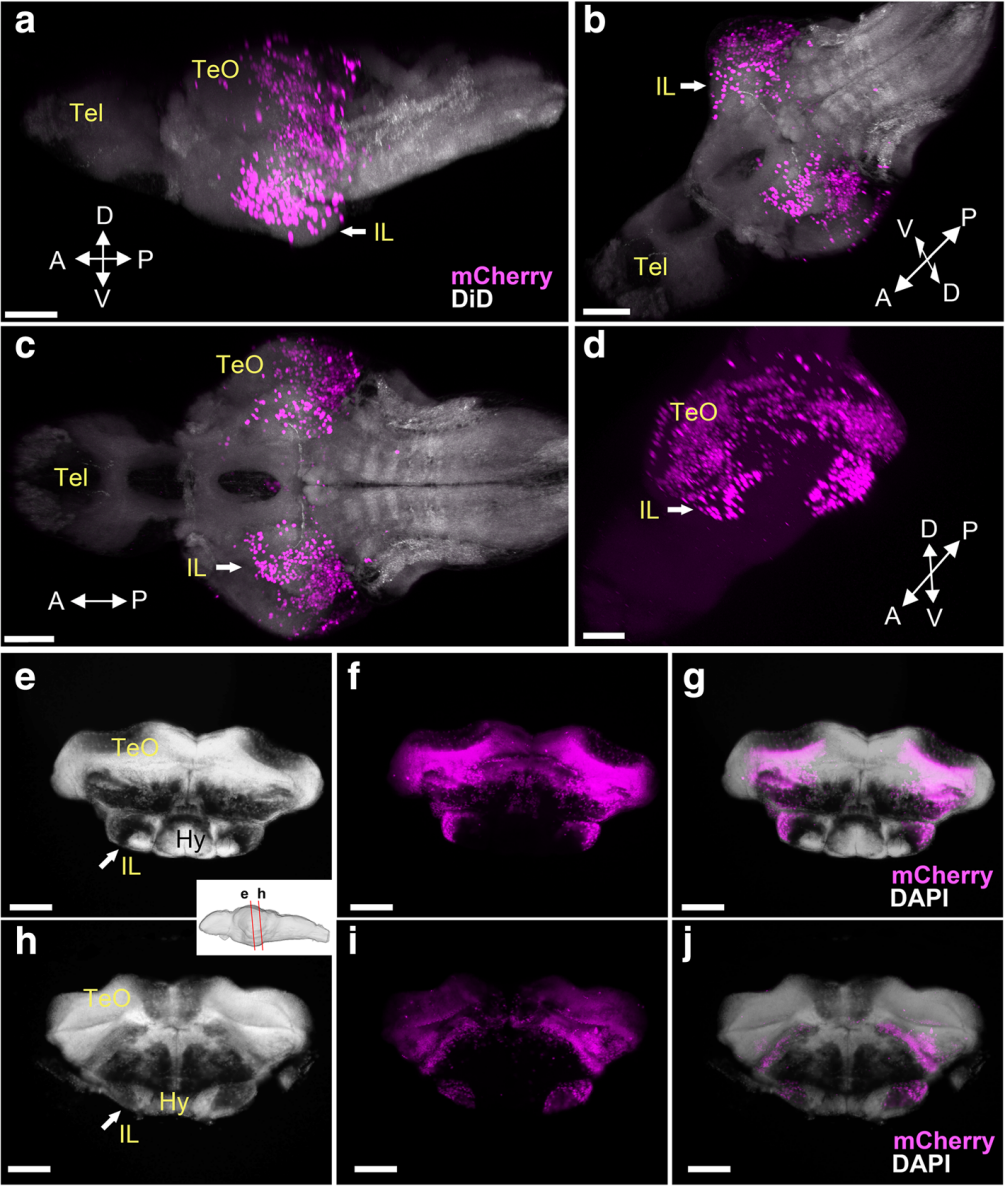
Localization of the mCherry-positive cells in late larval brains of *Tg(her5:ERT2CreERT2;βact:lox-stop-lox-hmgb1:mCherry)* zebrafish treated with tamoxifen at 24 hpf. **a**–**d** 3D reconstruction from confocal images of a 14 dpf brain, showing mCherry-positive cells in magenta (**a**–**d**) and DiD fiber labeling in gray (**a**–**c**). Four different views are presented: lateral (**a**), ventral (**c**), and two different obliques (**b**, **d**). Arrows point at the IL on one hemisphere. The IL bulging can be seen in **a** and **b**, while **d** displays the continuity of the mCherry-positive cells in the IL with other midbrain structures. **e**–**j** Frontal sections of a 19 dpf brain, showing mCherry-positive cells in magenta and DAPI nuclear labeling in gray. IL is clearly visible in frontal sections (arrows), at two different antero-posterior levels (indicated in sagittal view in the white box). Anteriorly (**e**–**g**) the mCherry-positive cells are on the lateral part of IL that appears continuous with more dorsal midbrain structures, while posteriorly (**h**–**j**) a cluster of the mCherry-positive cells is seemingly detached from the dorsal midbrain structures. At the posterior IL, most of the IL is mCherry positive. Scale bars, 80 μm. Abbreviations: Hy hypothalamus, IL inferior lobe, TeO optic tectum, Tel telencephalon


From late larval to juvenile stages, we could clearly identify the IL as a ventral protuberance with mCherry cells. Observation of global mCherry expression in the whole brain confirms a continuity of the mCherry labeling between the IL and more dorsal midbrain structures known to be part of the tectum and tegmentum (Fig. 5a–d, Additional file 4: Movie S3). This is also visible in frontal sections at 19 dpf, in which mCherry-positive cells form a continuous strip from the dorsal tectum to the ventral IL (Fig. 5f). At this stage, frontal sections of the IL already resemble those in adult, both anteriorly (Fig. 5e–g) and posteriorly (Fig. 5h–j). At 5 weeks post-fertilization (wpf), the IL continues to grow and appears as a ventral protuberance (Additional file 5: Figure S2).

Additional file 4:**Movie S3.** Localization of the mCherry-positive cells in the 14 dpf larval brain of *Tg(her5:ERT2CreERT2;βact:lox-stop-lox-hmgb1:mCherry)* zebrafish treated with tamoxifen at 24 hpf (3D visualization of Fig. 5a–d). mCherry-positive cells are shown in magenta, and DiD fiber labeling is shown in gray. (MP4 2602 kb)


### Formation of the IL in relation to the lateral recess

**Fig. 6:**
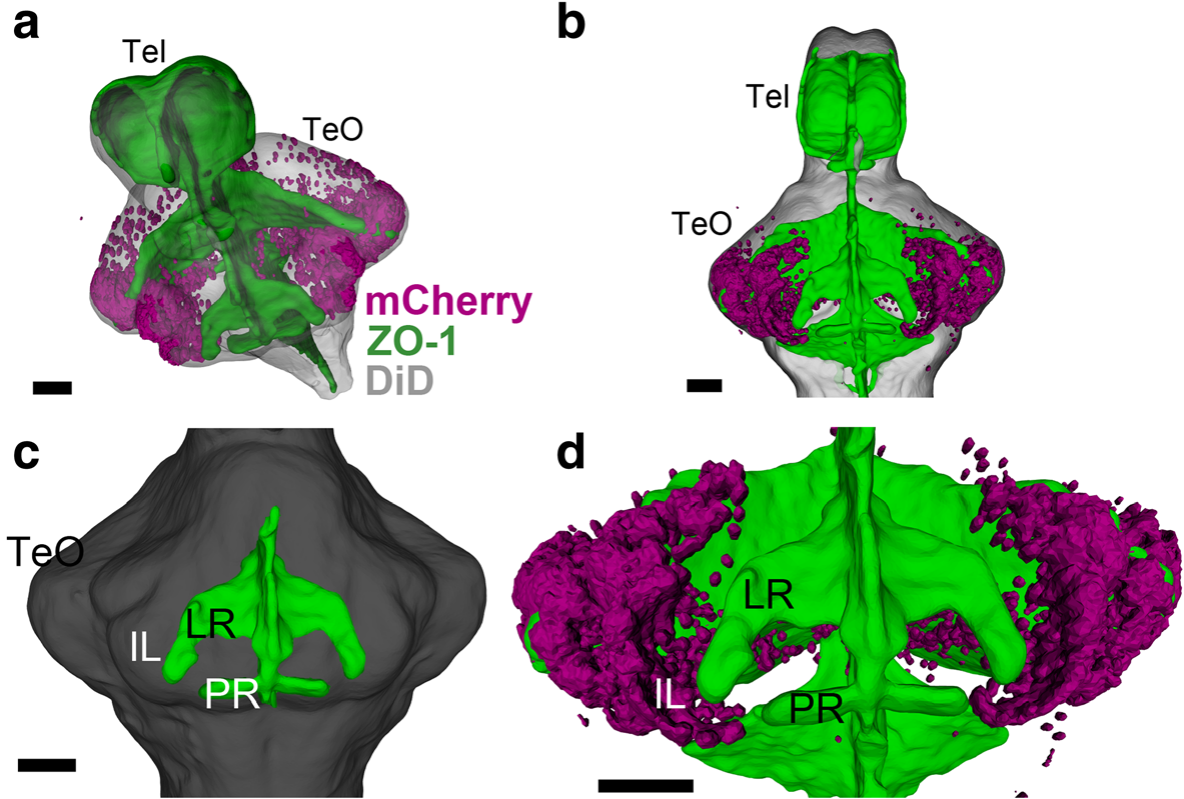
Developing IL in relation to the ventricular morphology. 3D reconstruction of image segmentation from confocal images of a 14 dpf zebrafish brain. ZO-1 (ventricular labeling) is shown in green (**a**–**d**), mCherry-positive cells are shown in magenta (**a**, **b**, **d**), and DiD fiber labeling is shown in gray (**a**–**c**). **a**, **b** Oblique (**a**) and ventral (**b**) views of the brain, showing the general distribution of the mCherry-positive cells in relation to the ventricular organization. **c** A ventral view highlighting the lateral recess (LR) and the posterior recess (PR) (anterior of the brain to the top). **d** A higher magnification of **b** focusing on the mCherry cells in relation to the LR. The mCherry cells are continuous from the tectal region, but they are devoid of proximity of the ventricular zone. Scale bars, 50 μm. Abbreviations: IL inferior lobe, LR lateral recess, PR posterior recess, Tel telencephalon, TeO optic tectum. The interactive version of this figure can be found at https://zenodo.org/record/2556246

In order to better understand how the IL is formed around the LR, we performed a 3D reconstruction of the mCherry-positive cells in relation to the ventricular morphology (Fig. 6a–d, https://zenodo.org/record/2556246). ZO-1 immunostaining labels tight junctions of neuroepithelial cells [17, 18]; thus, it can be used to visualize the ventricular zones of the brain.

Teleosts possess two distinct hypothalamic recesses, LR and PR, which are already observable at 48 hpf [4, 7]. A vast extension of the LR is found at later stages of development, and in the 14 dpf brain, the LR elongates in a posterior direction close to the PR (Fig. 6c, https://zenodo.org/record/2556246).

**Fig. 7:**
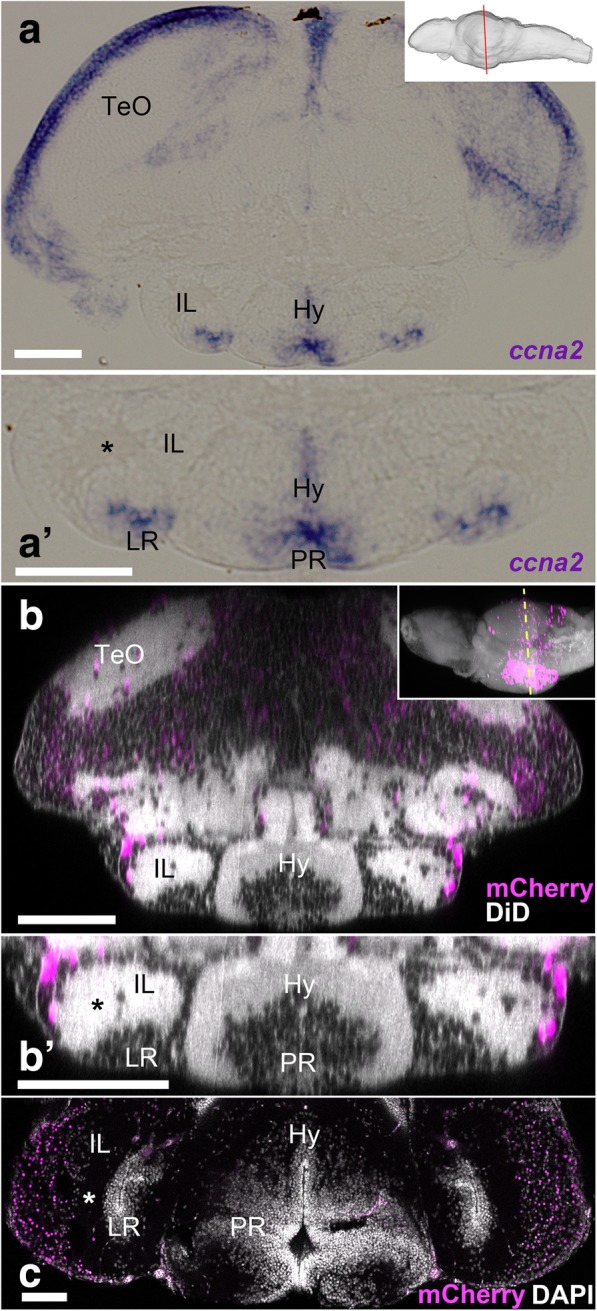
Comparison of juvenile and adult IL in zebrafish. **a**, **a’** Frontal section of the 14 dpf brain showing the transcripts of *ccna2*. The plane of the section is indicated in sagittal view in the right upper corner. **a’** A higher magnification of the ventral part of **a** containing IL. The expression of *ccna2* is found around LR. **b**, **c** Brains of *Tg(her5:ERT2CreERT2;βact:lox-stop-lox-hmgb1:mCherry)* zebrafish treated with tamoxifen at 24 hpf.  **b**, **b’** Frontal view of the 14 dpf brain which was obtained from 3D reconstruction of confocal images, showing mCherry-positive cells in magenta and DiD fiber labeling in gray. The plane of the section is indicated in sagittal view in the right upper corner. **b’** A higher magnification of the ventral part of **b** containing IL. **c** Frontal section of the adult IL showing mCherry-positive cells in magenta and DAPI nuclear labeling in gray. Note that the gray represents DiD fiber labeling in **b** and **b’**, while it corresponds to DAPI nuclear labeling in **c**. The asterisks indicate the cell-free fiber zone that separates the mCherry-positive external zone and the ventricular zone (around LR). The mCherry-positive cells are much abundant in the adult IL than in the larval IL. Scale bar, 90 μm. Abbreviations: Hy hypothalamus, IL inferior lobe, LR lateral recess, PR posterior recess, TeO optic tectum

3D visualization of mCherry-positive cells in relation to the ventricular zone clearly shows that the mCherry-positive cell cluster, which is continuous with the tectal region, covers the external part of the IL (Fig. 6a). In contrast, the ventricular zone of the LR is devoid of mCherry-positive cells (Fig. 6d, https://zenodo.org/record/2556246). In situ hybridization for *ccna2*, a cell proliferation marker, demonstrates that ventricular cells around the LR are in proliferation, whereas there is no proliferating cell in the external zone where mCherry-positive cells are found (Fig. 7a, a’). A closer look at the IL shows that the LR ventricular zone and the mCherry-positive external zone are separated by a cell-free fiber-rich zone (Fig. 7a’, b’, c; asterisks). Thus, IL is constituted of two anatomically distinct areas: the mCherry-negative ventricular zone is likely to be formed by the cells originating from the LR wall, while the mCherry-positive external zone is formed by the progeny of cells originating from the MHB.

Comparison between 14 dpf and adult IL (after induction at 24 hpf) shows that the relative size of the mCherry-positive external zone is significantly increased in the adult IL. At 14 dpf, there is only a thin layer of mCherry-positive cells (Fig. 7b’). In the adult (Fig. 7c), the mCherry-positive area is enlarged, forming a thicker mass laterally (which corresponds to the DIL). It is also worth noticing that the relative size of the whole IL is larger in adult and that the increase of the number of mCherry-positive cells largely contributes to the growth of IL mainly through its external portion.

### Later maturation of IL

**Fig. 8:**
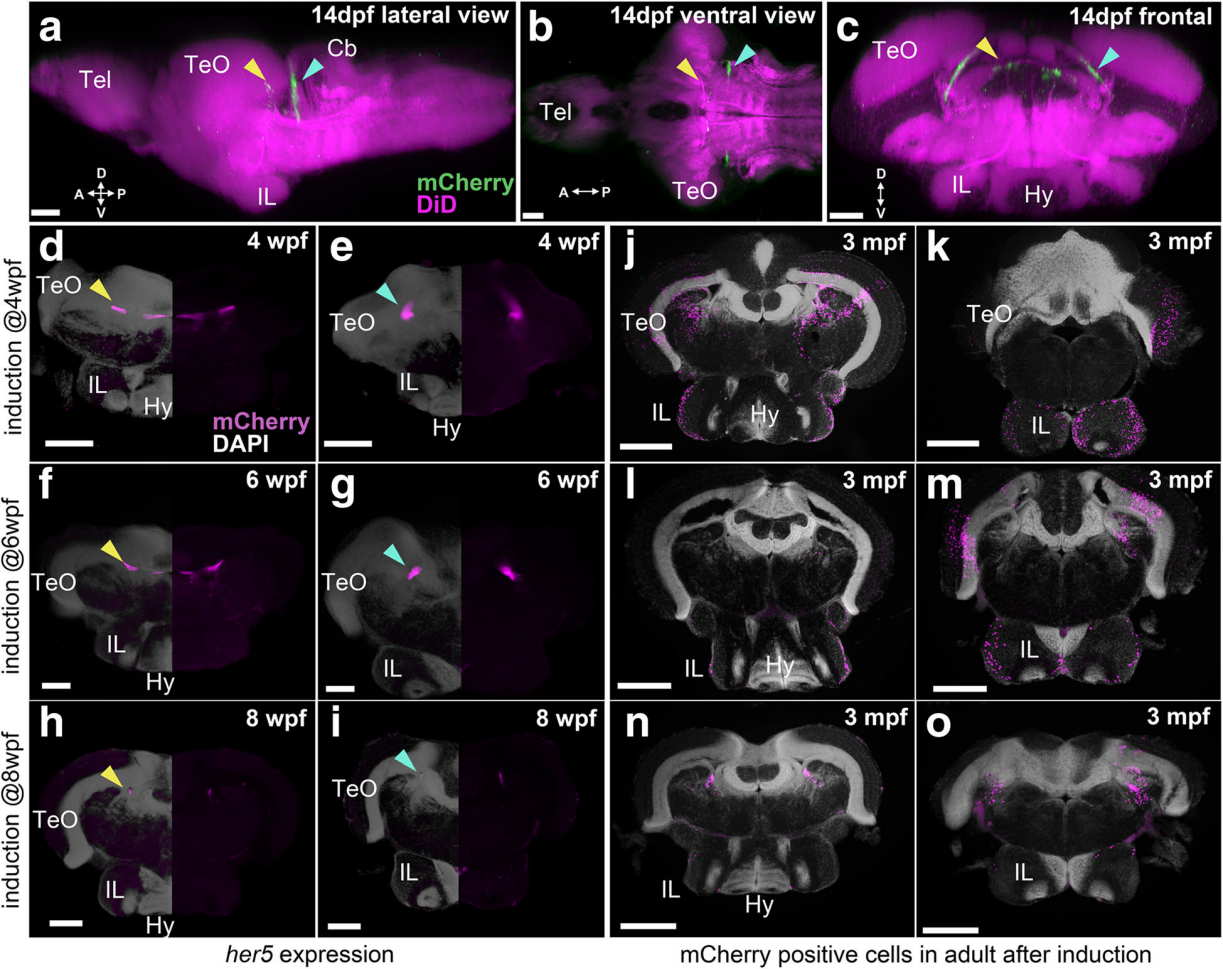
Localization of *her5* progenies following the induction at juvenile stages. **a**–**i** The brains of *Tg(her5:mCherry)* zebrafish to indicate the *her5* expression at late larval to juvenile stages. **j**–**o** The brains of *Tg(her5:ERT2CreERT2;βact:lox-stop-lox-hmgb1:mCherry)* to indicate their progenies in the adult (3 mpf) brains. **a**–**c** 3D reconstruction from confocal images of a 14 dpf brain, showing mCherry (representing *her5* expression) in green and DiD fiber labeling in magenta. Yellow arrows indicate the anterior *her5*-expressing domain, while blue arrows indicate the posterior *her5*-expressing domain. **a** The whole brain in lateral view, **b** in ventral view, and **c** in a frontal section from the same 3D visualization. **d**–**i** Frontal sections of juvenile brains of *Tg(her5:mCherry)* (**d**, **e** at 4 wpf, **f**, **g** at 6 wpf, and **h**, **i** at 8 wpf), showing mCherry (representing *her5* expression) in magenta and DAPI nuclear labeling in gray. The right half of the brain is demonstrated without DAPI to better visualize the mCherry signals. **d**, **f**, **h** The sections containing the anterior *her5*-expressing domain (yellow arrows). **e**, **g**, **i** The sections containing the posterior *her5*-expressing domain (blue arrows). **j**–**o** Frontal sections of 3 mpf brains of *Tg(her5:ERT2CreERT2;βact:lox-stop-lox-hmgb1:mCherry)*, after tamoxifen induction at the corresponding juvenile stages (**j**, **k** are the brain induced at 4 wpf, **l**, **m** at 6 wpf, and **n**, **o** at 8 wpf). **j**, **l**, **n** The sections showing the anterior IL. **k**, **m**, **o** The posterior IL. Note that the mCherry labelings (magenta) in these sections represent progenies of the cells shown in **d**–**i**. Scale bar: **a**–**c**, 50 μm; **d**–**g** 100 μm; **h**, **i** 200 μm; **j**–**o**, 350 μm. Abbreviations: Cb cerebellum, Hy hypothalamus, IL inferior lobe, Tel telencephalon, TeO optic tectum


The *her5* expression domain decreases in size during development but remains specific to the midbrain (Fig. 8a–c, Additional file 6: Movie S4 for 2 wpf; Fig. 8d, e, Additional file 7: Figure S3C for 4 wpf; Fig. 8f, g, Additional file 7: Figure S3F for 6 wpf; and Fig. 8h, i for 8 wpf). In the juvenile brain, *her5* is expressed only in two cell clusters along the tectal ventricular zone: one more anteriorly (Fig. 8; yellow arrowheads) and another more posteriorly (Fig. 8; blue arrowheads), which are considered to be the alar part of the mesencephalon. A short-term tracing experiment showed that a few days after the tamoxifen induction, a few induced mCherry cells were observed at the two mesencephalic locations, but not in the forebrain (Additional file 8: Figure S4).

Additional file 6:**Movie S4.** 14 dpf brain of *Tg(her5:mCherry)* zebrafish, with a focus on the cells expressing *her5* endogenously (3D visualization of Fig. 8a–c). For better visualization, the brain is truncated anteriorly in the middle of the optic tectum (TeO) and posteriorly in the cerebellum (Cb). The section includes about half of IL ventrally. mCherry-positive cells are shown in green, and DiD fiber labeling is shown in magenta. The movie starts in frontal view. When switching to lateral/dorsal views, anterior is on the left, posterior is on the right. (MP4 2500 kb)


Observation of their progeny at 3 months post-fertilization (mpf) confirms that they contribute to the formation of IL and that IL continues to grow until late juvenile stages. The *her5* progenies are restricted to the outer zone close to the surface of the IL (Fig. 8j–o). This suggests that cells are inserted at the periphery of the IL, all along development. Comparison of 3 mpf brains induced at 4 wpf (Fig. 8j, k), 6 wpf (Fig. 8l, m), and 8 wpf (Fig. 8n, o) clearly shows that induction at later stages results in less mCherry labeling. Thus, the growth of IL is slowing down over time, and IL is nearly mature around 8 wpf.

### Comparison between zebrafish and cichlid IL

**Fig. 9:**
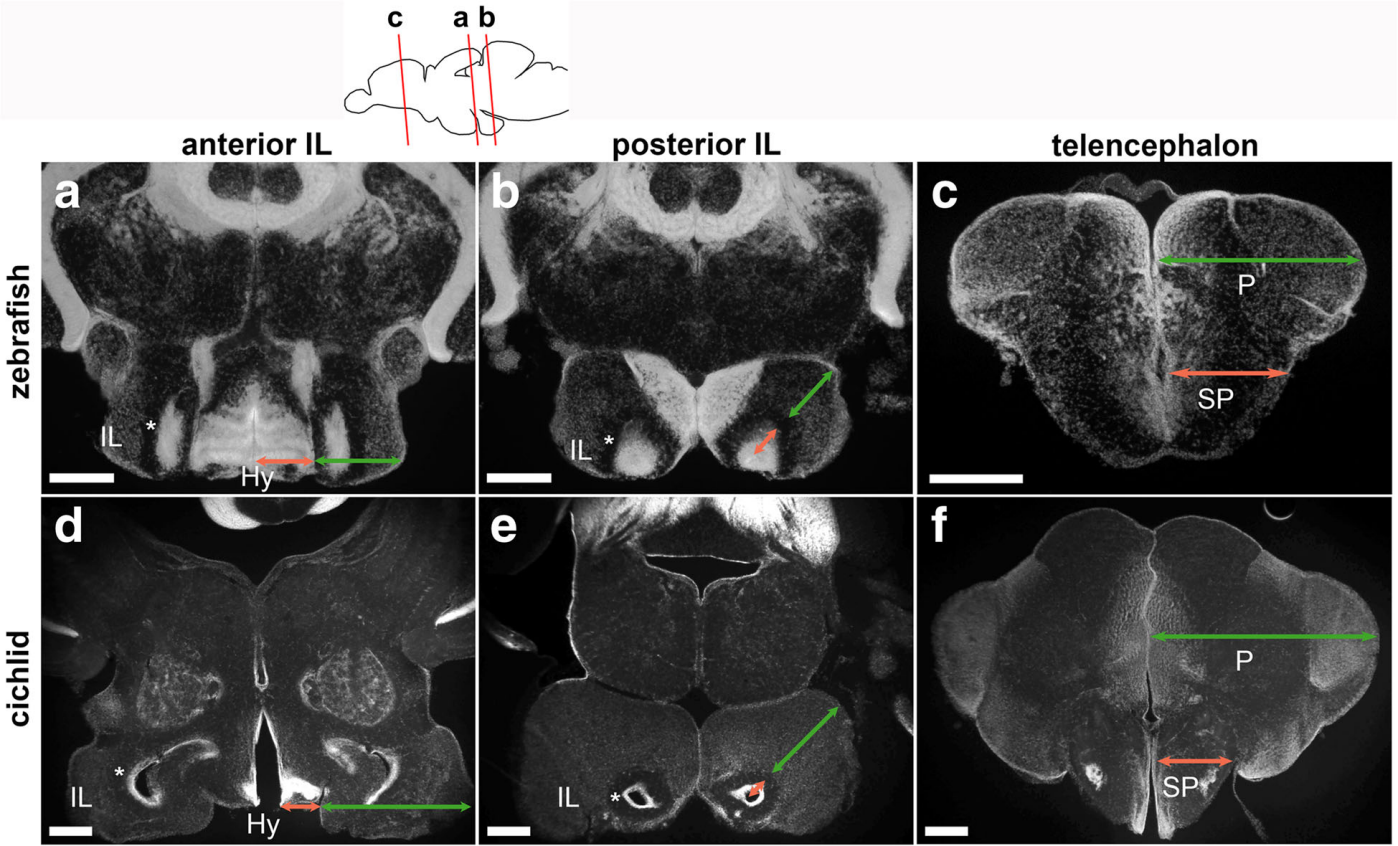
Comparison of the zebrafish and cichlid brains. Frontal sections of the brains of zebrafish (**a**–**c**) and cichlid (**d**–**f**), showing DAPI nuclear labeling in gray. The plane of the zebrafish sections is indicated in the schematic drawing on the top, and comparable level of the cichlid brain is shown below each zebrafish section. **a**, **d** Anterior IL. **b**, **e** The posterior IL. **c**, **f** The telencephalon. The relative size of the cichlid IL (**d**, **e**) is much larger than that of the zebrafish IL (**a**, **b**). It is prominent in comparison with the size of the hypothalamus (Hy; the size indicated in red arrows in **a** and **d**) that is located medial to the IL (the size indicated in green arrows in **a** and **d**). Also, the relative size of the external zone (the size indicated in green arrows in **b** and **e**) in comparison with the internal ventricular zone (the size indicated in red arrows in **b** and **e**) is much larger in cichlid. The asterisks (*) in **a**, **b**, **d**, and **e** indicate a cell-free fiber zone separating the external and internal zones. The relative size of the pallium (P; the size indicated in green arrows in **c** and **f**) in comparison with the subpallium (SP; the size indicated in red arrows in **c** and **f**) is much larger in cichlid than in zebrafish. Scale bar: **a**-**c**, 200 μm; **d**–**f**, 350 μm. Abbreviations: Hy hypothalamus, IL inferior lobe, P pallium, SP subpallium

The IL is a brain structure observed in all teleost species investigated so far. We compared the general organization of the IL of zebrafish with another teleost species, a Malawian cichlid (*Maylandia zebra*). As it is the case for the zebrafish, cichlid IL is also divided into a cell-dense ventricular zone along the LR and a cell-sparse external zone, which are separated by a cell-free fiber zone (Fig. 9; asterisks).

Nonetheless, there are significant differences between zebrafish and cichlid IL. The relative size of the IL is much larger in cichlid. This is obvious by comparing the IL (the size indicated in green arrows in Fig. 9a, d) with the caudal zone of the hypothalamus (Hy; the size indicated in red arrows in Fig. 9a, d) that is located medially. It is also clear that the proportion of the external zone (mesencephalic part; the size indicated in green arrows in Fig. 9b, e) in comparison with the ventricular zone (hypothalamic part; the size indicated in red arrows in Fig. 9b, e) is much larger in the cichlid IL.

Thus, the organization of the IL changes significantly between young and mature zebrafish brains and also between different species of teleosts. Note that in the cichlid brain, the relative size of the pallium (dorsal telencephalon) is also much larger than in the zebrafish brain (compare Fig. 9c, f, also see the “Discussion” section).

## Discussion

### New hypothesis on the developmental origin of IL

We took advantage of an inducible Cre transgenic line under the control of *her5* promoter to trace the progenies of cells originating from the MHB in zebrafish [13]. The *her5* transcription factor is known to be expressed in the MHB, corresponding to the midbrain primordium including both tectum and tegmentum [11, 12]. We followed the ontogeny of the induced mCherry-positive cells, from the earliest time point when the mCherry expression is visible (3 dpf). We confirmed that all the mCherry-positive cells are derived strictly from the MHB area, in other words, none is derived from the forebrain primordium. Thus, based on the cell lineage, we could conclude that all mCherry-positive cells found anterior to the MHB are of mesencephalic origin.

We have shown that PG and IL that were previously classified as forebrain are mainly composed of MHB progenies and therefore are actually mesencephalic. Our data showing a mesencephalic component for the PG shed a new light on the evolutionary scenario of ascending sensory pathways. The PG relays ascending sensory inputs to the pallium, and it has been compared to the dorsal thalamus (which is in the forebrain) in mammals [19]. Thus, our finding suggests that the teleost relay nucleus may not be homologous to the mammalian thalamus. The sensory pathways to the pallium would have evolved independently in mammals and teleosts.

**Fig. 10:**
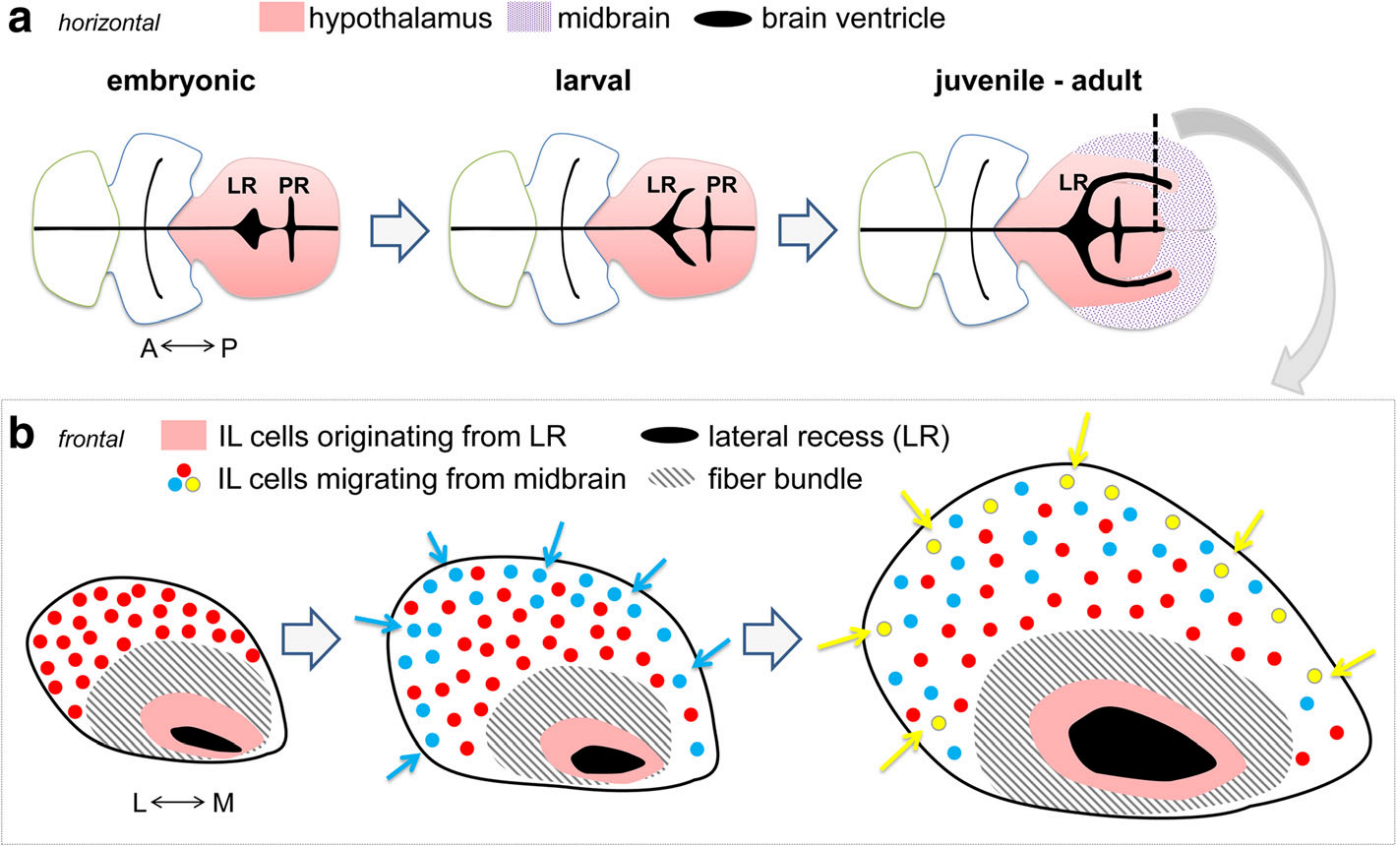
Schematic drawing summarizing development of IL. **a** Horizontal views of the zebrafish anterior brain, highlighting the development of the lateral recess (LR). LR elongates caudolaterally and covers the posterior recess (PR), forming IL. The ventricular zone of IL around the LR is hypothalamic (pink), while the external zone of IL is formed by cells migrating from the midbrain (purple dots). **b** Frontal views of IL showing maturation of the external zone of IL during juvenile stages. The external zone grows by insertion of cells originated from the midbrain. The red, blue, and yellow circles represent mesencephalic cells added at different time points during development

The IL has been considered to be a part of the teleostean hypothalamus (thus forebrain). This structure appears as a caudal continuation of the hypothalamus forming an additional “lobe.” There is no equivalent structure in tetrapods or in ray-finned fishes such as *Polypterus* and sturgeon; thus, IL would have evolved only in the teleostean and holostean fishes (*Neopterygii*). Our data suggest IL is formed by cell populations that have different developmental origin: the LR ventricular zone, which is hypothalamic (mCherry-negative), and the external zone, which is mesencephalic (mCherry-positive). The presence of proliferating cells around the LR suggests that the internal ventricular zone is generated by progenitors along the LR. Considering that the LR is an elongation of the hypothalamic ventricle, we can conclude that the mCherry-negative ventricular zone is hypothalamic (Fig. 10a). By contrast, we postulate that the external zone of IL is generated by migrating cells from the tectal ventricular zones, since there is no sign of proliferation in the external zone of IL. The continuous stream of mCherry-positive cells from the dorsal tectal area to the ventral IL suggests that the mesencephalic cells invade a brain structure that grows from the anterior “hypothalamic” LR towards posterior.

Interestingly, IL maturates progressively until the late juvenile stage (summarized in Fig. 10b). Our cell counting of mCherry-positive cells (Additional file 2: Figure S1) indicates that at least half of the progenitors of external IL cells would be generated around 24 hpf (since not all the cells would recombine), and the rest of IL progenitors would be progressively generated during the larval-juvenile stages.

The cells are added at the periphery all along development, with less cells added the older the fish is. The newer cells do not seem to form new layers stacked upon the previous ones, but intercalate with preexisting cells. If successive layers were apposed on each other, mCherry-positive cells induced at 4 wpf should be located in a deeper layer than those induced at 6 wpf (compare Fig. 8k, m). Instead, we always observe the mCherry-positive cells at the surface. Thus, IL seems to grow by adding newer cells inserted between the existing cells on the surface.

### Functional implication of IL

Considering that a large part of IL is not hypothalamus, the interpretation of IL functions needs also to be reconsidered. Even though the periventricular part is hypothalamic, our previous studies have already demonstrated that the “hypothalamus” of teleosts and mammals is very different and that careful comparative analyses are required before concluding a simple one to one homology [4, 5, 7]. For example, we have previously shown that a large part of the teleost hypothalamus is mainly composed of a cell type (CSF-contacting neurons) that has been secondarily lost in placental mammals [7]. The hypothalamic CSF-contacting populations are particularly increased in teleosts, around the additional hypothalamic recess (posterior recess, PR) that evolved specifically in teleosts. Thus, the teleost hypothalamic functions cannot be inferred in a simple manner based on available mammalian data. IL would be another example showing the particularity of the teleost brain.

Functions of IL were initially suggested by a set of studies of electrical stimulation of the brain in freely moving fish. Demski and his colleagues showed that electrical stimulation of IL evoked movements such as biting at mirror or snapping objects [20, 21]. IL is also known to receive gustatory sensory inputs [22–24]. These data were interpreted with the assumption that IL is hypothalamic, and this is why IL has been proposed to be involved in feeding behaviors [14, 25].

A recent study using zebrafish larvae has shown that IL is activated by visual detection of moving objects [26]. The activation of IL and chasing behavior were evoked by presenting moving objects other than food, such as a moving spot on a screen. Nonetheless, the interpretation was that IL is involved in feeding motivation, because IL was assumed to be homologous to the lateral hypothalamus of mammals. Without this preconception of homology, the data by Muto et al. (2017) simply suggest the involvement of IL in visual detection, and there is no data concerning the motivational state. Indeed, their results rather favor another hypothesis proposing that IL is involved in sensory integration [24, 27–29].

Based on connectivity data in several teleost species, IL receives various sensory inputs in addition to gustatory: visual, somatosensory, auditory, and probably lateral line. Thus, IL has also been proposed to be a multisensory integration center [24, 27–29]. Taking the electrostimulation data into consideration, IL may integrate multimodal sensory information and evoke motor responses. In the zebrafish larval brain, neuronal connectivity is not fully established, and stimulus-response association is rather simple: visual detection of small moving objects evokes chasing and biting, which is synonymous with feeding behavior.

In the mature brain, notably in teleost species like cichlids, the sensory-motor integration should be more complex. Some species of cichlids and wrasses demonstrate behavioral repertories such as nest construction or tool use [30–32]. Based on available anatomical data, these families have a large IL as well as a large pallium [33, 34], being consistent with our observations (Fig. 9d–f). IL is known to receive efferent projections from the pallium in various species [24, 27, 29, 35, 36]. In this context, anatomical and functional correlation between the pallium and IL is an interesting issue to be examined.

## Conclusion

Our findings have revealed that some structures which have been considered as part of the forebrain are actually mesencephalic in the zebrafish brain. This refines the current model of the brain regionalization. In addition, the revision on the regional identity of IL modifies the interpretation of previous studies concerning its function. Zebrafish has become an important model in neuroscience. In order to interpret data correctly, we have to be careful with the interpretation of the homology between structures in teleosts and mammals.

## Methods

### Animals

Zebrafish (*Danio rerio*) used in this study were raised in our own colony. Zebrafish embryos and larvae cannot be categorized as male or female. For adult zebrafish, both sexes were used and there was no significant difference between male and female. For *her5* and *ccna2* in situ hybridization, wild-type (AB) embryos (*n* = ~ 30) were used. For studying the lineage tracing of *her5*-expressing progenitors (*n* = ~ 100 for 24 hpf and ~ 10 for each experiment of 3–8 wpf), we crossed two transgenic lines previously used in a recent publication [13]: *Tg(her5:ERT2CreERT2)* and *Tg(βact:lox-stop-lox-hmgb1-mCherry)* (see tamoxifen treatment for details). For visualization of *her5* expression, we also used another transgenic line (*n* = ~ 30) *Tg(her5BAC:nls-mCherry*^*gy3*^*)* that is simply referred to as *Tg(her5:mCherry)* [13]. Embryos/larvae up to 5 days post-fertilization (dpf) were maintained and staged as described previously [37]. After larval stages, zebrafish were raised in our fish facility. Three months post-fertilization (3 mpf) or older zebrafish is considered as adult.

Juvenile Malawian cichlids (*Maylandia zebra*) were kindly provided by Dr. Joël Attia (Neuro-PSI, Université de Lyon/Saint-Etienne). Three brains (2 females and 1 male) were used in this study, and there was no difference in the general cytoarchitecture of IL between male and female.

### Tamoxifen treatment

Tamoxifen treatments were performed in double transgenic fish generated by crossing *Tg(her5:ERT2CreERT2)* and *Tg(βact:lox-stop-lox-hmgb1:mCherry)* as described previously [13, 38]. 4-Hydroxytamoxifen (Sigma-Aldrich T176) was dissolved in ethanol at a concentration of 10 mg/ml and stored at − 20 °C until use. The working solution was freshly prepared before the treatment, further diluted with embryo medium (for 24 hpf) or fish water (for 4–8 wpf). The animals were incubated in the tamoxifen working solution at 28 °C in the dark.

24 hpf embryos were dechorionated with Pronase (Sigma-Aldrich P5147; 1 mg/ml) prior to the tamoxifen treatment. Embryos were placed into the six-well culture plate (Thermo Fisher Scientific) and were incubated with 10 μg/ml tamoxifen for 6 h. After the incubation, the fish were rinsed three times with embryo medium, then put back to the incubator.

For juvenile stages (4–8 wpf), fish were placed in a beaker (100–200 ml fish water depending on the number of fish) with an air pump and incubated with 2 μg/ml tamoxifen on four consecutive days. The incubation time per day was 4 h, but the treatment was interrupted whenever the fish looked sick. At the end of each incubation, the fish were gently rinsed three times with fish water, placed back to a clean fish tank, and fed.

The tamoxifen-induced mCherry expression was observed at 3 mpf, except for the experiment of short-term tracing. In case of the short-term tracing (Additional file 8: Figure S5), the fish was sacrificed 4 days after the end of the tamoxifen treatment (that is 1 week after the beginning), and immunofluorescence anti-dsRed (see below) was performed to observe the mCherry expression.

### Tissue preparations

Zebrafish embryos at 24 hpf and 3 dpf were fixed in ice-cold 4% paraformaldehyde (PFA; Electron Microscopy Sciences) in 0.01 M phosphate-buffered saline (PBS; Fisher Scientific) containing 0.1% Tween20 (PBST) overnight at 4 °C. Zebrafish older than 5 dpf were deeply anesthetized using 0.2% tricaine methanesulfonate (MS222; Sigma-Aldrich) diluted in fish water. The fish were fixed in 4% PFA in PBST overnight at 4 °C, then brains were dissected out. Zebrafish embryos used for in situ hybridization (ISH) were dehydrated in ethanol gradient series and kept at − 20 °C in methanol at least for a couple of days. They were rehydrated prior to ISH. For immunolabeling, samples were conserved in a stocking solution containing 0.5% PFA and 0.025% sodium azide. Brains were sectioned in a frontal plane (80 μm) with a vibratome (Leica VT 1000 S). Tissue clearing was performed for whole-mount imaging of zebrafish embryos or larvae (see below).

Cichlid brains were obtained from juvenile individuals (body size around 6–8 cm). The animals were deeply anesthetized in 0.2% MS222 diluted in water and transcardially perfused with cold 4% PFA in PBS. Brains were dissected, post-fixed with 4% PFA overnight at 4 °C, then conserved in the stocking solution until use. Brains were sectioned in a frontal plane (80 μm) with a vibratome.

### Tissue clearing

Considering the relatively small thickness of zebrafish brains at larval stages, a simplified clearing protocol was applied as described in Affaticati et al. (2018) [39]. Depigmentation step was applied as follows: up to 15 larvae were incubated in 10 mL of pre-incubation solution in a petri dish (0.5× saline sodium citrate buffer (SSC), 0.1% Tween20) for 1 h at room temperature without stirring. Then, samples were bleached by incubation in depigmentation solution (0.5× SSC, 5% formamide, 3% H_2_O_2_). Samples were left in the solution until pigments were completely degraded. Samples were then washed three times in PBST and left overnight in PBST.

### Immunofluorescence

Immunofluorescence on larval zebrafish brains (14 dpf and earlier) was performed in 2 mL glass vials. Thorough PBST washes were performed between each antibody incubation step. The samples were incubated at room temperature for 5 h in a blocking solution containing 10% normal goat serum (NGS), 10% dimethyl sulfoxide (DMSO), 5% PBS-glycine 1 M, 0.5% Triton X-100, 0.1% deoxycholate, and 0.1% NP-40 in PBST. Samples were then incubated in staining solution (2% NGS, 20% DMSO, 0.05% sodium azide, 0.2% Triton X-100, 1× PBS, 0.1% Tween20, 10 μg/ml heparin) with anti-dsRed (1:600; Clontech Laboratories 632496, RRID: AB_10013483, Lot# 1612022) at room temperature for 3–4 days with gentle shaking, on a 3D rocker.

Double immunolabeling for dsRed and ZO-1 (1:100; Invitrogen 33-9100, RRID: AB_87181, Lot# SA241427) was performed in 14 dpf brains. Samples were incubated with secondary antibodies conjugated to fluorophores (1:600) in staining solution at room temperature for 3–4 days with gentle shaking. Alexa Fluor 546 goat anti-rabbit (Thermo Fisher Scientific A-11010, RRID: AB_143156, Lot# 1733163) was used for dsRed, and Alexa Fluor 488 goat anti-mouse (Thermo Fisher Scientific A-11001, RRID: AB_2534069, Lot# 1572559) was used for ZO-1. At the end of the second day of the secondary antibody incubation, DiD (Invitrogen/Thermo Fisher Scientific L7781; 1 μg/ml) was added for membrane labeling, while YOYO-1 (Molecular Probes/Thermo Fisher Scientific Y-3601; 200 nM) was added for nuclear labeling. Finally, samples were incubated in a fructose-based high-refractive index (RI) solution that is adjusted to 1.457. This solution was obtained as described in Affaticati et al. [40].

For brain sections of the adult individuals induced at 24 hpf, mCherry endogenous fluorescence was bright enough to allow direct imaging. For other brain sections, anti-dsRed immunofluorescence (1:600 in PBST containing 4% NGS and 0.3% Triton X-100) was performed. The sections were incubated with the primary antibody at 4 °C overnight and then with secondary antibody Alexa Fluor 546 (1:1000 in PBST) at 4 °C overnight. In order to visualize the brain morphology, the sections were counterstained with DAPI (4′,6-diamidino-2-phenylindole dihydrochloride; Sigma-Aldrich; 5 μg/ml) at room temperature for 15 min.

### In situ hybridization (ISH)

cRNA probes of zebrafish *her5* [13, 41], *ccna2* [4, 42], and *ert2Cre* [13, 43] were provided by Dr. L. Bally-Cuif (Pasteur Institute, Paris, France). ISH were performed in toto for 24 hpf zebrafish embryos, while on brain sections for animals older than 14 dpf. Detailed ISH procedures have been described in our previous publications [4, 7].

After rehydration, the 24 hpf embryos or brain sections were first incubated in hybridization buffer at 65 °C for 4 h and then hybridized with 2 ng/ml of cRNA probe in hybridization buffer at 65 °C for at least 18 h. Samples were then washed in gradient series of formamide/2× SSC mixture at 65 °C: 75% formamide/25% 2× SSC, 50% formamide/50% 2× SSC, 25% formamide/75% 2× SSC, then washed in 2× SSC and finally in 0.2× SSC. After being rinsed with PBST at room temperature, the samples were incubated with anti-digoxigenin conjugated with alkaline phosphatase (1:2500; sheep anti-DIG-AP Fab fragments; Roche Diagnostics 11093274910, RRID: AB_514497, Lot# 12486522) at 4 °C overnight. After PBST washes, the signal was visualized by incubation with nitroblue tetrazolium chloride (NBT) and 5-bromo-4-chloro-3-indolylphosphate (BCIP) solution (Roche Diagnostics 11681451001) in 0.1 M Tris-HCl (pH 9.5)/0.1 M NaCl in H_2_O (TN buffer). The 24 hpf embryos were embedded into 3% agarose, sectioned with a vibratome in a sagittal plane (40 μm), and slide mounted.

### Image acquisition

A Leica TCS SP8 laser scanning confocal microscope was used to image adult sections with a × 25 water immersion objective. For tissue-cleared in toto specimens, the same microscope was used with a Leica HC Fluotar L × 25/1.00 IMM motCorr objective. For all these acquisitions, fluorescence signal was detected through laser excitation of fluorophores at 405, 488, 552, or 638 nm and detection was performed by two internal photomultipliers. Steps in the *Z*-axis were fixed at 1 μm. Epifluorescence images were acquired using a Multizoom AZ100 (Nikon). Bright-field images were either acquired with an upright microscope BX43 (Olympus) or the Multizoom. Acquired images were adjusted for brightness and contrast using ImageJ/FIJI software.

### Quantification of mCherry-positive cells in IL

The mCherry-positive cells in the adult external IL were counted from confocal images using the ImageJ cell counter module. We used stacks of 10 μm frontal sections at anterior and posterior levels, obtained from two specimens of independent experiments of inductions at 24 hpf. The total number of cells was determined with DAPI nuclear labeling of the external IL, that is excluding the ventricular (inner) portion of and corpus mamillare (CM).

### 3D image reconstruction

Whole specimens (3 dpf entire head; 3, 5, 7, and 14 dpf entire brain) imaged in confocal microscopy were reconstructed in 3D using Imaris 8.0.1 software (Oxford Instrument Company) using the “3D view” visualization tool on a Dell T3610 workstation.

Image segmentation was performed interactively in the segmentation editor of Amira 6.0.1 (FEI Company) on a Dell T630 running Ubuntu 16.04. In preparation of the segmentation of the ventricle volumes, the raw data of the ZO1 signal was cleaned up by subtracting the mCherry signal: [ZO1]-[mCherry]. Similarly, the raw data of the mCherry signal were cleaned up before segmentation by weighted subtraction of the reference channel (DiD): [mCherry]-([DiD]/10). These steps remove low-level contamination (DiD signal bleed-through) with the subtracted signal (mCherry or DiD respectively) from the signal of interest (ZO1 or mCherry), and by this facilitate thresholding and reconstruction. The ZO1 signal was isolated using a combination of local thresholding (Magic Wand) and manual segmentation (Brush) along all three cardinal axes of the data’s coordinate system. For coping with the thin, sheet-like shape of the ventricles, we dilated the segmented volume by two voxels and smoothed it in 3D (three times with a mask size of 6). The mCherry signal was segmented from the cleaned-up data by global thresholding and smoothed in 3D with a mask size of 3. The resulting surfaces were generated with Amira’s “Generate Surface” module (Smoothing: Existing Weights), simplified to about 180K faces each, using Amira’s Simplification Editor and exported to the “Polygon File Format” (ply) for the visualization in other software tools.

## Additional files

Additional file 2: **Figure S1.** Quantification of mCherry-positive cells in the external IL. Proportion of mCherry labeled cells (gray bar) in relation to the total number of DAPI-labeled cells was calculated from frontal sections of adult zebrafish brains (tamoxifen induction at 24 hpf). The section level of “anterior” corresponds to the level of Fig. 3b, and the level of “posterior” corresponds to the level of Fig. 3c. The “total” represents sum of them. (PDF 51 kb)

Additional file 5: **Figure S2.** Localization of the mCherry-positive cells in the 5 wpf juvenile brain of *Tg(her5:ERT2CreERT2;βact:lox-stop-lox-hmgb1:mCherry)* zebrafish treated with tamoxifen at 24 hpf. Frontal sections showing mCherry-positive cells in magenta and DAPI nuclear labeling in gray. A-C show the anterior IL and D-E show more posterior IL. Scale bars: 100 μm. Abbreviations, Hy: hypothalamus, IL: inferior lobe, TeO: optic tectum. (TIF 10750 kb)

Additional file 7: **Figure S3.** Endogenous expression of *her5* in juvenile zebrafish brains. In situ hybridization of *her5* on frontal sections of 4 wpf (A-C) and 6 wpf (D-F) brains. The plane of each section is indicated in the schematic drawing on the top. There is no *her5* expression in the anterior sections containing forebrain regions (A, B, D, E). In the brain sections containing the mesencephalic region, *her5* expression is found along the tectal ventricular zone (C, F; arrows). Scale bar: 100 μm. Abbreviation, Di: diencephalon, IL: inferior lobe, Tel: telencephalon, TeO: optic tectum, Tg: tegmentum. (TIF 17072 kb)

Additional file 8: **Figure S4.** Short-term tracing of tamoxifen-induced mCherry-positive cells in the *Tg(her5:ERT2CreERT2;βact:lox-stop-lox-hmgb1:mCherry)* juvenile zebrafish brain. Frontal sections of a 4 wpf brain, showing mCherry-positive cells in magenta and DAPI nuclear labeling in gray. The plane of each section is indicated in the schematic drawing on the top. A, B Anterior brain sections containing forebrain regions where there is no mCherry-positive cell. C, D More posterior brain sections containing mesencephalic regions where a few mCherry-positive cells are found close to the tectal ventricular zone. C’ and D’ show the area squared in C and D at a higher magnification. Scale bar: 60 μm for A and B, 100 μm for C and D, and 10 μm for C’ and D’. Abbreviation, Hy: hypothalamus, IL: inferior lobe, PG: preglomerular nucleus, Tel: telencephalon, TeO: optic tectum, V: ventricle. (TIF 18788 kb)
