# Supplementary figures and images for: Mesencephalic origin of the inferior lobe in zebrafish
Source: BMC Biol. 2019 Mar 8;17:22. doi: 10.1186/s12915-019-0631-y (PMC6407210; doi:10.1186/s12915-019-0631-y)

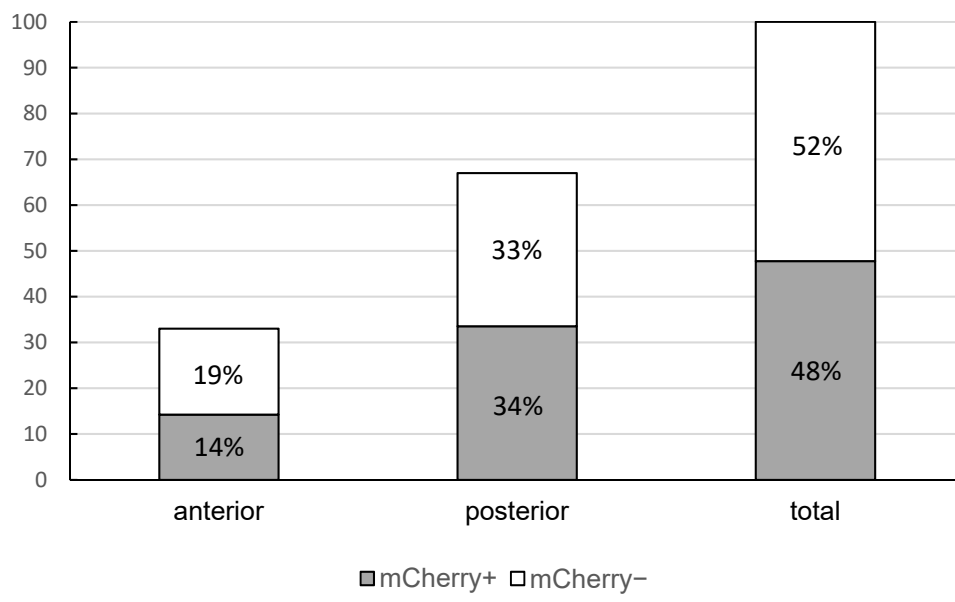

Supplement: Supplementary file 5 — Figure S1. Quantification of mCherry-positive cells in the external IL. Proportion of mCherry labeled cells (gray bar) in relation to the total number of DAPI-labeled cells was calculated from frontal sections of adult zebrafish brains (tamoxifen induction at 24 hpf). The section level of “anterior” corresponds to the level of Fig. 3b, and the level of “posterior” corresponds to the level of Fig. 3c. The “total” represents sum of them. (PDF 51 kb) [file 12915_2019_631_MOESM2_ESM.pdf]

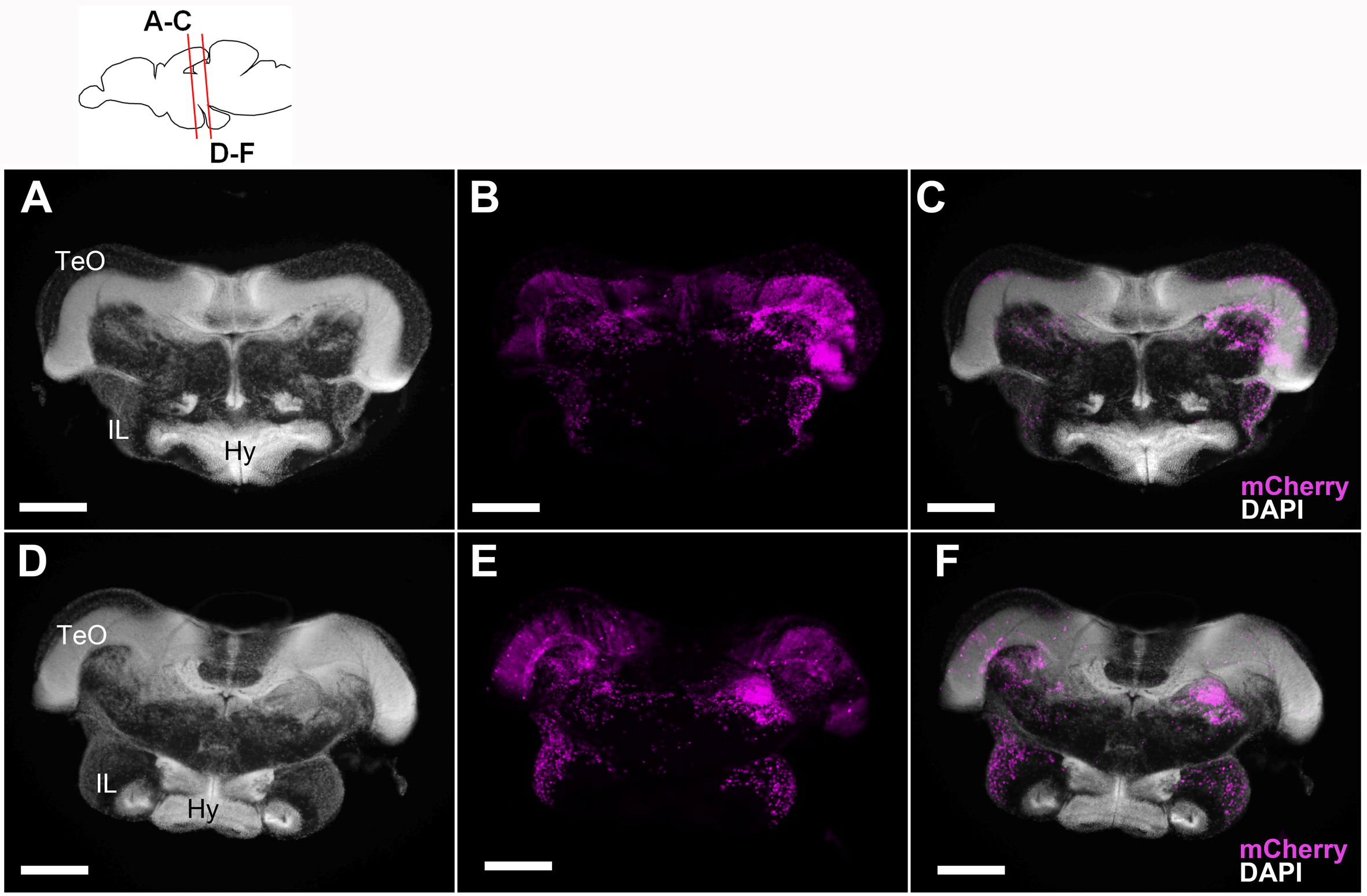

Supplement: Supplementary file 6 — Figure S2. Localization of the mCherry-positive cells in the 5 wpf juvenile brain of Tg(her5:ERT2CreERT2;βact:lox-stop-lox-hmgb1:mCherry) zebrafish treated with tamoxifen at 24 hpf. Frontal sections showing mCherry-positive cells in magenta and DAPI nuclear labeling in gray. A-C show the anterior IL and D-E show more posterior IL. Scale bars: 100 μm. Abbreviations, Hy: hypothalamus, IL: inferior lobe, TeO: optic tectum. (TIF 10750 kb) [file 12915_2019_631_MOESM5_ESM.tif]

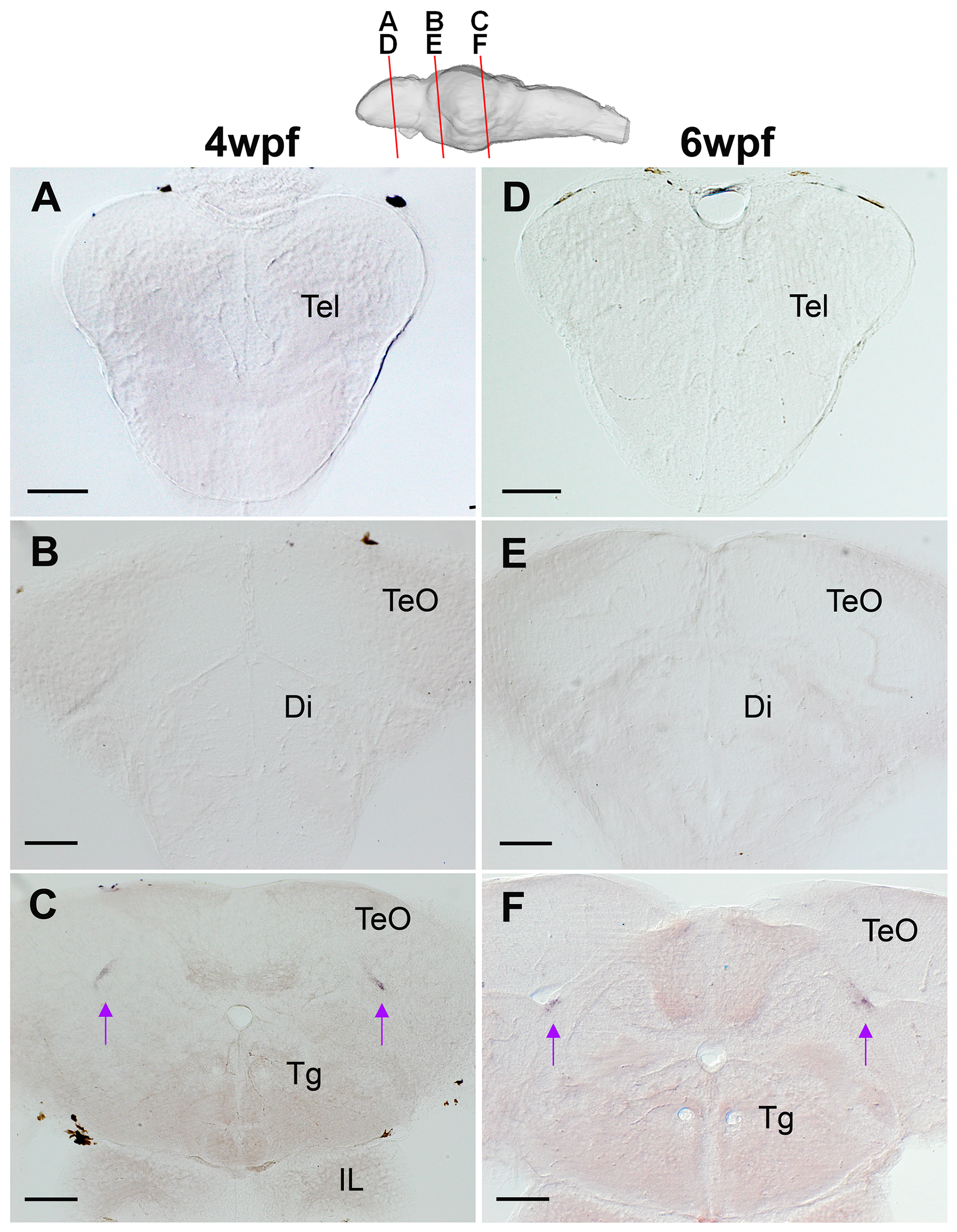

Supplement: Supplementary file 7 — Figure S3. Endogenous expression of her5 in juvenile zebrafish brains. In situ hybridization of her5 on frontal sections of 4 wpf (A-C) and 6 wpf (D-F) brains. The plane of each section is indicated in the schematic drawing on the top. There is no her5 expression in the anterior sections containing forebrain regions (A, B, D, E). In the brain sections containing the mesencephalic region, her5 expression is found along the tectal ventricular zone (C, F; arrows). Scale bar: 100 μm. Abbreviation, Di: diencephalon, IL: inferior lobe, Tel: telencephalon, TeO: optic tectum, Tg: tegmentum. (TIF 17072 kb) [file 12915_2019_631_MOESM7_ESM.tif]

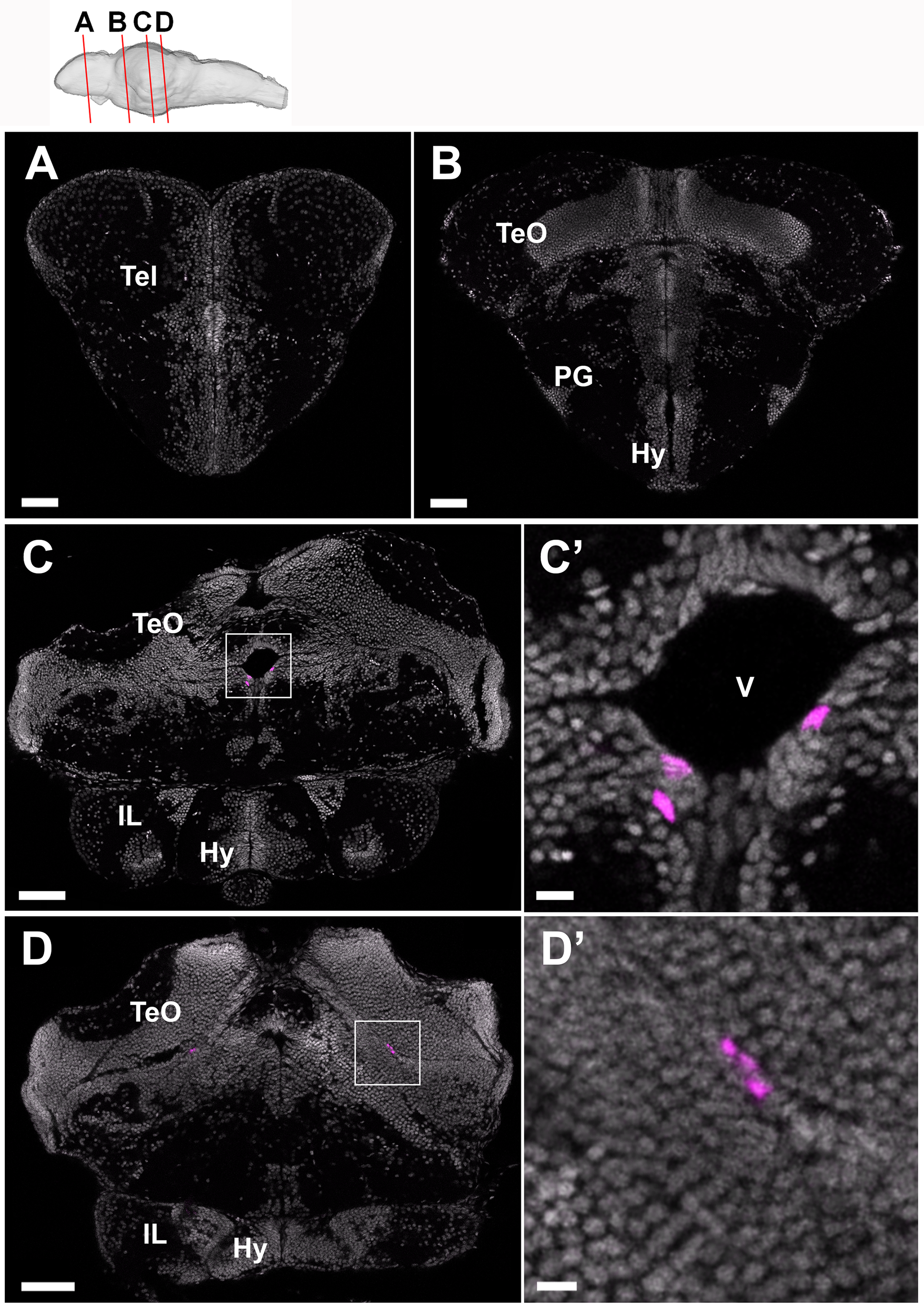

Supplement: Supplementary file 8 — Figure S4. Short-term tracing of tamoxifen-induced mCherry-positive cells in the Tg(her5:ERT2CreERT2;βact:lox-stop-lox-hmgb1:mCherry) juvenile zebrafish brain. Frontal sections of a 4 wpf brain, showing mCherry-positive cells in magenta and DAPI nuclear labeling in gray. The plane of each section is indicated in the schematic drawing on the top. A, B Anterior brain sections containing forebrain regions where there is no mCherry-positive cell. C, D More posterior brain sections containing mesencephalic regions where a few mCherry-positive cells are found close to the tectal ventricular zone. C’ and D’ show the area squared in C and D at a higher magnification. Scale bar: 60 μm for A and B, 100 μm for C and D, and 10 μm for C’ and D’. Abbreviation, Hy: hypothalamus, IL: inferior lobe, PG: preglomerular nucleus, Tel: telencephalon, TeO: optic tectum, V: ventricle. (TIF 18788 kb) [file 12915_2019_631_MOESM8_ESM.tif]
